# Virtual Clinical Listening Groups for Psychological Intervention With University Students in the COVID-19 Pandemic

**DOI:** 10.3389/fpsyt.2022.772698

**Published:** 2022-06-03

**Authors:** Lucas Bloc, Juliana Lima de Araújo, João Marcos Leite, Sarah Rebeca Barreto, Karla Carneiro, Anna Karynne Melo, Georges Boris, Virginia Moreira

**Affiliations:** ^1^Postgraduate Program in Psychology, University of Fortaleza (UNIFOR), Fortaleza, Brazil; ^2^Laboratório de Psicopatologia e Clínica Humanista-Fenomenológica (APHETO), Fortaleza, Brazil; ^3^Postgraduate Program in Public Health, University of Fortaleza (UNIFOR), Fortaleza, Brazil; ^4^Graduate in Psychology, University of Fortaleza (UNIFOR), Fortaleza, Brazil

**Keywords:** COVID-19, listening groups, university students, psychological intervention, mental health

## Abstract

The pandemic has affected people's mental health and university students are considered one of the most vulnerable groups, encouraging the development of psychological interventions that can minimize the suffering and illness of this public. Among the possibilities of intervention, virtual clinical listening groups were created which, during the period of social isolation, had the purpose of opening up a listening space for university students suffering from emotional distress. The focus of the group meetings was how university students were experiencing the pandemic, as well as the consequences of this experience. In this article, we discuss virtual clinical listening groups as a form of psychological intervention in the mental health care of university students during the COVID-19 pandemic at a university in northeastern Brazil. This is a qualitative study that began with 274 university students and 5 psychologists who facilitated the listening groups. We used as instruments the registration forms, the participation records of the virtual clinical listening groups and the field diaries written by the facilitators after each meeting. We described in the results the collected data and, from the field diaries, the themes that emerged in the various meetings were listed and analyzed in dialogue with the literature. At the end of the psychological screening process, 117 students participated in the 17 organized groups, with an average of 8 university students per group. Among the main motivations for participating in virtual clinical listening groups, we highlight: the desire to share experiences, a search for control of anxiety, depression and stress, care for mental health, a willingness to promote listening to psychological support for themselves and for others, and self-knowledge. We discuss the nuances in the process of forming the listening groups and the characteristics of the participants, as well as a central element of the group process, which is the establishment of bonds and mutual help among the participants. Feeling vulnerable and the fear of contamination is an element of suffering and, above all, of stress experienced by university students. The group presented itself as an alternative of mutual care in the pandemic context.

## Introduction

Pandemics imply psychosocial disturbances that can exceed the population's ability to cope, both with the disease itself and with factors adjacent to it, such as possible generalized panic, anxiety and stressful situations. In the case of the pandemic caused by the new Coronavirus (COVID-19), this situation can be accentuated by the fact that the disease is still recent, dynamic and plural, due to the numerous mutations that the virus has undergone. According to statistics from the World Health Organization; WHO (2020), COVID-19 can cause an increase in the incidence of mental disorders in around one third to half of the affected population (1).

Although physical health and fighting disease are the primary focus of attention, mental health is a fundamental factor to be observed during the pandemic and post-pandemic. It is considered that the psychological implications may be longer lasting and extend far beyond the current period of the COVID-19 pandemic, echoing in different sectors of society (2, 3). The pandemic, with all the aspects that it involves, from social distancing to vulnerability in the face of imminent contamination, directly affects on a large scale people's mental health. We are experiencing the biggest public health emergency in decades, which demands a more effective and broader look at mental health.

Some factors are considered to be a greater risk for mental health in the pandemic, such as being a woman, being a student, and having physical symptoms or previous health problems linked to COVID-19, all associated with higher levels of anxiety, depression and stress (4). The development of psychological interventions is a fundamental strategy to minimize the negative implications and promote mental health, in addition to being a significant element in the readaptation process in view of the losses and changes that have been taking place (2, 3, 5–7). Considering that the pandemic has affected people's health in general, psychological interventions can minimize the implications for mental health resulting from COVID-19. As a result of this new scenario, a training and learning process in higher education is necessary in order to train professionals with skills and competences in this new and future context (8).

Psychological interventions should be diversified and, at first, focus on stressors related to the disease, in this case, COVID-19, and on the difficulty in adjusting to life due to the restrictions in the period of distancing (9). Thus, any proposal for more immediate intervention must address issues experienced in the present and with the aim of reducing or preventing psychological difficulties in the long term. Thus, psychological interventions can promote mental health, enhance a new adaptation to the world and favor the creation of strategies to deal with the losses and the necessary transformations due to post-traumatic stress and financial losses.

The mental health of students and their adaptation to the University are themes that were widely discussed even before the pandemic caused by COVID-19 and, from its beginning, researchers found that some students began to experience even more severe crises (10). Given this problematic situation, some recent researches carried out in Universities discuss university life and the mental health of its students (2, 5–8, 11). As a form of intervention, some Brazilian Universities developed projects and programs aimed at the wellbeing of their students by promoting actions of listening and psychological counseling, psycho-pedagogical guidance and psychotherapeutic referral (8, 12, 13). In the Brazilian public sector, Universities, through the National Student Assistance Program (PNAES) (14), guarantee housing, food, transport, health care, digital inclusion, culture, sports, daycare, and pedagogical support for their students. Even with these commendable actions, in the pandemic context, it must be recognized that student demands did not disappear along with the suspension of on-site academic activities. These demands continue to permeate the universe of students, who require answers as urgent as their daily problems. The complexity of the pandemic in the university context requires the development of new strategies to face inequalities in higher education, aimed at meeting academic demands, which have intensified with the pandemic (8, 12, 13).

The focus of this article is on one of the possible forms of psychological intervention, those carried out in groups, which, in their clinical nature, can function as collective spaces for health care. As they can lead to greater relational exchange and more interpersonal cooperation by expanding the possibility of expressing feelings and greater openness to one's own experience and to the other, (15) we believe in their preventive character, not just as treatment as it occurs in the psychotherapy group.

Based on this idea, in the first half of 2020, during the COVID-19 pandemic, virtual clinical listening groups were created at a university in Fortaleza, Brazil, offering qualified psychological listening in the first great wave of the pandemic for people who underwent emotional suffering, investing in the clinical potential of qualified listening in the university context.

In their preventive nature, groups can serve a larger number of students, strengthening their autonomy and enabling each participant to develop self-care strategies based on the encounter with the other. Clinical group listening can also function as a means of building paths that promote significant changes for all group participants. The virtual listening groups facilitated by the psychotherapists developed interventions focused on the present, bringing to the surface the difficulties experienced by the groups' participants themselves. As these students have had a shared university experience, virtual listening groups can be a more precise form of short-term mutual care by gathering participants coming from the same context (15–17). Thus, interaction with other people who experience similar processes can be an advantage made possible through the modality of intervention in groups on individual psychological care, as it allows closer contact with other experiences, creating new possibilities in terms of support and mutual care (16, 17).

This article aims to discuss virtual clinical listening groups as a form of psychological intervention in the mental health care of students during the COVID-19 pandemic at a university in Northeastern Brazil. These groups, carried out remotely during the period of social distancing, had the objective of listening to university students with different psychological demands, related to how they experienced the COVID-19 pandemic and its consequences in their lives. They were used as a type of mental health promotion and care in a crisis context through the sharing and discussing of common themes and experiences in the lives of the virtual clinical listening group participants during the pandemic.

## Materials and Methods

This is qualitative study (18) that describes and discuss the phenomena that emerged in the groups from the field diaries prepared by the facilitators after the completion of each group.

### Participants and Study Site

The research was carried out by a team of Psychology professionals at a large private university located in the city of Fortaleza, a reference in the north and northeast of Brazil. Due to the pandemic and considering the modality of remote classes at the University, the entire research was carried out virtually, from its dissemination through the University's marketing to the realization of clinical listening groups.

Participants in this research made up a total of 279 adults, 274 of which were group participants and five facilitators who, in addition to coordinating the virtual clinical listening groups, recorded them in field diaries. As for the group participants, we established being an undergraduate or graduate student at the University of Fortaleza aged 18 years or older as the inclusion criterion. Students who presented demands diverging from the virtual listening group proposal, a clinical picture of considerable emotional instability, or a psychological profile indicating the need for individual psychological or psychiatric care in the triage interview were excluded. In these cases, upon being identified, they received the appropriate referral to professional services consistent with their needs. The sample of facilitators was composed of psychologists with experience in group work and who were also part of the research team.

### Instruments

The data collection instruments used by the research team were: registration forms (completed on the Google Forms platform by the university students) and field diaries (written after each meeting by the research team using the Google Docs platform), which provided descriptions about the group's dynamics and the themes that emerged in the meetings.

In the registration, the general number of registered participants and those who confirmed their participation after the psychological screening and actually attended the meetings of the virtual clinical listening groups were registered. Field diaries were written right after the group session in order to contemplate as much as possible everything that had emerged in the group. These records focused, above all, on the experience of the COVID-19 pandemic from the university students perspective.

### Data Collection Procedure

Data collection took place from September to November 2020. Results were computed in November 2020. Initial data refer to registration information obtained through the Google Docs form, prior to the completion of the virtual clinical listening groups. The psychological screening and group follow-up processes were registered weekly by the team in the Google Forms spreadsheet during the research period.

Each team of psychologists responsible for facilitating virtual clinical listening groups contacted previously registered university students through the WhatsApp messaging tool and scheduled psychological screening interviews. In these clinical interviews, carried out by video call on Google Meet, the expectations and motivations of university students interested in participating in the group were raised; their doubts were also clarified; and the interviewers, through clinical listening, assessed whether the participation of the university student in the groups could contribute to their mental health and that of the other participants, or whether there was any demand that required another type of intervention. This triage process was a fundamental step in the composition and functioning of the virtual clinical listening groups, by identifying, above all, the participants' demands and the students' current psychological state. The psychotherapists used their clinical experience to identify potentially psychopathological experiences that would render their participation in the group inappropriate. If doubts or the need for a more accurate diagnostic understanding arose, the cases were discussed by the research group to reach a more effective clinical decision. Cases contraindicated for virtual clinical listening group participation were referred to health care services and/or professionals in the city. In addition, the terms of the psychological contract were reinforced: the importance of guaranteeing confidentiality and the need for continuous use of the audio and video equipment in a safe environment. The Free and Informed Consent Form (FICF) was also presented. After confirming the completion of the terms, the facilitator forwarded the link to the room where the virtual clinical listening group would take place on the day and time the student enrolled.

The clinical listening groups always took place in the remote/online modality through the Google Meet platform. This online platform was chosen because students were already familiar with it due to its previous use in University classes. Each group lasted 2 (two) h and was facilitated by 2 psychologists, with a maximum of 15 students. In all, the team was made up of 5 facilitators from the virtual clinical listening groups, who rotated at various times and shifts to ensure greater student adherence.

Facilitation of the virtual clinical listening groups adopted a humanist-phenomenological perspective. (15, 17). In this sense, the facilitators incentivized the virtual clinical listening group by clarifying the group's purpose: to enable the development of a listening space for the participants' experience in the context of the pandemic. For 2 h, the issues brought by the virtual clinical listening group participants were worked, always keeping in mind the uniqueness of each experience, but considering the common aspects lived by the other group participants as well. There was no prior planning, that is, the needs and demands brought by the group itself were worked through. The facilitators, taking a phenomenological stance, sought to understand the meanings of the virtual clinical listening group participants' lived experience by providing them with a welcoming, non-judgmental environment.

At the end of the group sessions, each facilitator wrote a field diary about the group that had just been held, recording the themes that emerged in the students' experiences, as well as a description of the interaction between them. The itinerary of the group's participants is indicated from the following flowchart:

**Table:** 

Dissemination of groups at the University	Group registration	Screening process	Submission of the FIFC for signature and referral for participation in the group

For the team members, the flowchart was as follows:

**Table:** 

Identification of potential participants for screening based on the data provided by the registration form	Screening with enrolled students to define participants	Realization of the virtual clinical listening group	After the end of the group, writing the field diary

### Data Analysis

At the end of the project, all records and information collected in the instruments of this research were read. In an effort to record the virtual clinical listening group participants' data, the analysis was restricted to one description of the participants. The data collected *via* the Google Forms Platform was automatically entered into the system itself to help the research team access the group participants' descriptions.

From the field diaries prepared by the facilitators, after each group session, the themes that emerged in the various meetings were listed and analyzed in dialogue with the literature. The analysis of the material was carried out on a phenomenologically-focused basis (17). It sought to capture the facilitators' experience by describing the themes that emerged in the virtual clinical listening groups which were recorded by them after each meeting with the group. A phenomenological reduction tactic was adopted in the data analysis for understanding the meanings of the group participants' lived experience, starting with the suspension of their a priori knowledge about the conduct of clinical listening groups and seeking to learn what had arisen in the participants' experience before any theoretical elaboration.

The field journals enabled the elaboration of the lived experience's meaning in facilitating the groups, producing new knowledge around the weekly experience in the virtual clinical listening groups. Analysis of the descriptions of the experience recorded in the journals was done based on a phenomenologically critical method (17). Initially, the primary text was divided into movements, highlighting the verbal and non-verbal content that arose. From there, a descriptive analysis was developed with the aim of understanding the virtual clinical listening group phenomenon based on the description of its participants' experience, not on a priori knowledge. Finally, a dialog with the literature was constructed, which took into account the multiple contours intersecting these group participants' experience.

The themes identified and analyzed in this discussion reflect the participants' experiential dynamic through the lens of those who facilitated the virtual clinical listening groups. The descriptive analysis enabled a humanistic-phenomenological interpretation of the most immediate experience lived by the group in crisis, potentially providing for intervention into and promotion of mental health.

### Ethical and Legal Aspects of Research

The research followed the Resolutions 466/2012 and 510/2016 of the National Health Council, which deal with ethics in research involving human beings. This research was approved by the Research Ethics Committee of the University where the research was carried out, being registered under ruling number: 4,244,587.

## Results

With regard to the dissemination of the research and virtual clinical listening groups, 19 students were informed by indication from teachers, 38 by colleagues, 26 by email, 50 by social networks, 2 through the University's website and 198 by text message (text message received directly from the higher education institution's website or application).

Thus, 332 applications from university students interested in participating in virtual clinical listening groups were registered, these consisted of 273 women (83%) and 59 men (18%), 321 (97%) from undergraduate courses and 11 (3%) from post-graduate courses. Of this total, 26 applications were repeated by students who intended to participate more than once in the groups, and 4 re-enrolled because the group they intended to participate in did not occur due to low demand on the planned day and time. This totaled 30 re-enrollments. With regard to undergraduate courses, there was a predominance of two courses. Psychology (*n* = 168) and Law (*n* = 58). The others were distributed throughout various courses, such as. Administration, Architecture, Physiotherapy, Nursing, and Nutrition, among others, with regard to post-graduate courses, courses in psychology and public health were registered. At the end of enrollment, 302 students from the various courses of the aforementioned University were registered.

The psychological screening stage in the formation process of the virtual clinical listening groups, consisted of individual interviews with the aim of evaluating the students' psychological expectations and conditions to participate in the groups, in addition to clarifying their functioning. A total of 274 university students interested in participating in the groups were screened, while 8 dropped out of participation before the psychological screening and 20 did not respond to the team's attempts to contact them, totaling 28 dropouts.

Of the 274 university students screened, 120 were not recommended for participation in the virtual clinical listening groups: 37 did not accept the participation rules (such as turning on the camera, for example), 1 was a minor, 14 intended to participate only to do academic work, 32 withdrew from participation after psychological screening, 6 were University professors, not students, and 30, ~10%, as evaluated by the team of psychologists, were not considered to be in sufficient emotional condition to participate in virtual clinical listening groups, being referred to other psychological services (individual psychotherapy, psychiatric monitoring, etc.). In the latter cases, the team analyzed that, especially given the current pandemic, the groups, due to their punctual nature (only 2 h of meetings), would not meet the urgent demand of these students.

At the end of the psychological screening process, 154 registered university students were considered able to participate in the virtual clinical listening groups. At the end of the psychological screening process, 117 students participated in at least one (some more than one) of the 17 groups carried out, with an average of 8 university students per group. Table 1 above summarizes the data described below:

**Table 1:**
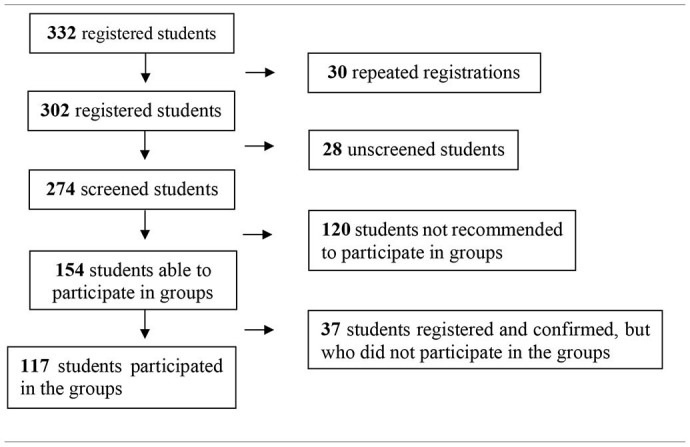
Number of university students enrolled, screened and participating in virtual clinical listening groups.

From the data collected in the registration forms and in the psychological screening of university students interested in participating in virtual clinical listening groups, it was found that the topic of mental health is still surrounded by ignorance and stigma, given that the large number of students considered able to participate in the groups (154) contrasted with the also considerable number of dropouts and absences after confirmation of participation (37). However, the university students who participated in the registered virtual clinical listening groups expressed recognition that such groups functioned as an area of expansion for students by providing access to channels in regard to care and discussion of mental health at the University itself. Among the main motivations for participating in virtual clinical listening groups, described by the university students in the registration forms and in the psychological screening, were: desire to share experiences; seeking to control anxiety, depression and stress; mental health care; intention to learn and study due to identification with group work; willingness to promote listening to psychological support for themselves and others; desire to help and to receive help; try to learn to deal with one's emotions; need for speech and acceptance; search for self-knowledge; difficulty in adapting during the pandemic period and seeking a greater understanding of the lived moment; attempt at time management, aiming for greater productivity; and desire to contribute to the research.

In addition to the motivations initially described in the registration forms and in the psychological screening, more comments emerged during the virtual clinical listening groups, such as: family experiences; situations related to motherhood, self-care and care for others; concern with mental health; effort to be well; feelings of malaise, irritation and anxiety; issues relating to academic life; difficulty with distance learning (ODL); interruption of life projects, need for productivity and difficulties at work; deprivation, loneliness, fear of virus contamination and loss during the pandemic; uncertainties; grief; and feelings of lack of control and vulnerability. Such motivations and statements reflect the difficulties that emerged and/or intensified in the experience of university students in the promotion of health and care for other people, which may be a reflection of the fact that the vast majority of university students participating in virtual clinical listening groups are students from the Psychology Course at the University where the research was carried out.

Starting from a phenomenological analysis that was performed as described in the Method Section, an initial reading of the field diaries was done to extract the topics presented in each diary. These topics were grouped in categories based on their common aspects and, above all, those that revealed the lived experiences in the listening groups. It is important to stress that the instrument was used by the group facilitators who, soon after the group session, described the experience of being in the group, noting the elements present. The set of topics became four categories.

In the first category, the central topic was the process of forming the groups, revealing the difficulty of doing group work and managing it virtually. The differences between the psychotherapists was also revealed in the diaries, as the sessions were always by two psychotherapists and the pairs also changed. The second category exposed a fundamental aspect: the bonds created and the mutual help that arose in the groups. Aspects such as group adhesion, quality the participants' presence, acceptance of the other, and genuine interest were described by the psychotherapists. Care for oneself and others, enabling the building of a dialog and, more importantly, a bond emerged in multiple diaries of various groups. Furthermore, when they were able to identify similar difficulties, the participants had the perception that they were not alone.

Stress, to be discussed here in the third category, was revealed as a common axis of suffering upon exposing the participants' vulnerable state. The participants were able to recognize how susceptible they were to the risks of the pandemic, experiencing anxiety at the possibility of personal projects being interrupted. As for college, they described the difficulty of keeping up with remote classes and conflicts with family members arising from cohabitation exacerbated by social isolation. In the fourth category is care, a fundamental aspect that may be experienced both as the sense of caring for another and that of being cared for. The pandemic revealed to the participants the need for listening. In the groups, they felt welcomed by their peers and by the psychotherapists, helping them to reveal experiences of grief, building bonds, and even the desire for continuity. It is worth noting that a large part of the participants expressed the need to create spaces for listening at the university.

## Discussion

### The Formation of Virtual Clinical Listening Groups

The development of psychological interventions during the pandemic period has been shown to be necessary due to the wide emotional impacts resulting from this international public health emergency. In this context, many people who already felt psychologically vulnerable saw their mental health worsen due to the wide changes and limitations in their daily lives resulting from the pandemic (5). The need for urgent psychological interventions in a collective context led to the option of using online group work due to its greater capacity to bring together more people in a shorter period of time. It is in this context that the virtual clinical listening groups were able to be not only an instrument used in the investigation of the mental health of university students during the current COVID-19 pandemic, but also a resource for access and psychological intervention in the mental health of the research participants herein analyzed.

It is important to emphasize that online psychological services have several advantages, especially in critical periods, such as the current COVID-19 pandemic. Among these advantages, they above all stand out as an important resource for coping with the social distancing caused by such an international public health emergency (19). As it was possible to observe in this research, this type of psychological intervention enables the confluence of speaking and mutual listening by university students who experience, on the one hand, common experiences in the face of the COVID-19 pandemic and, on the other, their own particular ways of coping.

Difficulties in the formation of virtual clinical listening groups may be associated with the lack of familiarity of university students with this type of intervention, their possible resistance to psychological care due to lack of information or even prejudice, and the minimization of the importance of mental health as a relevant aspect of their lives. The motivation to participate in these groups came mainly from the suffering resulting from the pandemic period. The focus on mental health proposed in these groups placed all the participants in a common field of experience. Although each participant expressed the particularity of their experience, all group participants sought out each other's experiences due to the suffering directly or indirectly associated with the pandemic (20). It can be confirmed that, in psychotherapeutic groups, regardless of their modality, such as virtual clinical listening groups, mutual support is developed among the participants as they can share anxieties, conflicts and various other experienced situations (21). Therefore, such spaces for exchange contribute to the processes of personal (8, 15, 16), interpersonal and group growth due to the development of cooperation among the participants (16).

It is important to highlight the role of screening in this type of psychological intervention. Commonly, psychological screening is aimed at collecting data, raising diagnostic hypotheses and defining the type of care that the person(s) need (22). With regard to virtual clinical listening groups, psychological screening had the fundamental role of identifying whether there was convergence between what university students were looking for and the virtual psychological service offered and, above all, of evaluating the psychological conditions of students enrolled to participate in a group with other students. A broader diagnostic understanding of the current situation of these students' mental health, that is, the level of suffering and illness they experienced, was essential to create favorable conditions for the group and contribute to the students' mental health. In addition, the moment of psychological screening was essential for facilitators and group participants to get to know each other beforehand, creating a favorable climate for listening and psychological support during the groups. Also in the screening, in order to guarantee more security regarding eventual psychological crises, the indication of a next of kin for emergency contact was requested, in case of complications.

Aiming at guaranteeing confidentiality in the virtual clinical listening groups, the conditions for the functioning of the groups were explained in the screening of each university student, and these conditions for the group were also reinforced at the beginning of each meeting with the group participants.

As it is a service offered by the University, the institution played a central role in disseminating the project to offer virtual clinical listening groups to its students and in their subsequent adherence. The more the University recognizes the impacts of the COVID-19 pandemic, the more it is able to develop precise and immediate preventive and treatment strategies for its students (5, 23, 24). The team of facilitators faced the challenge of discovering how to encourage and mobilize students from several different courses to participate in virtual clinical listening groups, which was one of their main difficulties. Communication is a central element in this process and it was possible to observe that the vast majority of students learned about the groups from a direct means of communication at the University, the text message, which demonstrates the importance of the participation of the higher education institution in this context of an international public health emergency. It is noteworthy that, historically, psychological and/or psychiatric services are underutilized (25, 26), contrasting with their current growing demand.

It is also important to emphasize that the modalities of psychological care offered at universities are quite diverse (29). In the context of the investigated University, there is an offer of practices and services aimed at students' mental health, which also involve the various aspects of their learning processes. It is noteworthy that the pandemic greatly changed the scenario and significantly affected the students, who, abruptly, lost a series of references that exist in the academic environment, which often demands a difficult adaptation and causes several obstacles in their mental health.

The virtual clinical listening groups showed that the high levels of stress, anxiety and depressive symptoms presented by university students are related, among others, to uncertainties regarding various aspects of their lives, including the educational environment, concerns with technology and online classes in their courses, social distancing, reduced family income, loss of family, friends and close people, as well as with their professional future. Such impacts are present in Universities around the world (3, 30). It is important to note that Brazil, among the 21 countries surveyed in the Global Student Survey (35), is the country with the highest rate in terms of the impact of the pandemic on mental health: 76% of Brazilian university students interviewed declared that the pandemic impacted their mental health. This study also points out that only 21% of them sought help, which corroborates with the research on virtual clinical listening groups analyzed here, where there is the difficulty of caring for their own mental health and the formation of mutual care groups. Although there is a high recognition for the need for psychological counseling and mental health care, it still seems to be frequently neglected among university students.

### Bonding and Mutual Help in Groups

The construction of the bond between psychology facilitators and university students participating in virtual clinical listening groups developed from the first contact through text messages. As highlighted by Sansom-Daly and Bradford (31), in health services performed online, it is essential to use a safe and accessible platform, and there should be a previous meeting in which it is clarified what to do in the case of technical difficulties and also to become familiar with the technological tool before using it. One of the challenges of virtual psychological interventions has been the greater likelihood of misunderstandings and miscommunications due to the reduced context in relation to face-to-face communication (32), which reinforced the need for attention to the communication of goals and how virtual clinical listening groups work.

As in the face-to-face process, during the realization of the virtual clinical listening groups, it was necessary that the facilitators were aware of elements that favor the interpersonal and group encounter, such as the quality of presence, attention, acceptance of the other, interest, care about themselves and others, the capacity for dialogue, spontaneity, congruence, among other important aspects (15–17). The feeling of psychological wellbeing tends to grow and strengthen when bonds are formed with other people, given the human need to build lasting interpersonal relationships. When such links are broken or modified, there is often significant psychological suffering (35).

The welcoming provided in the virtual clinical listening groups enabled the participating university students, even in a limited space of time, to share intimate experiences that caused them suffering. Group work made it possible for their self-support to be strengthened when they realized that they were not alone and that other people also experienced similar difficulties (15–17). The assurance that shared experiences would be kept confidential was another fundamental aspect so that students participating in the groups could be more available to experience them more fully and intensely. In the online space, boundaries tend to be looser, which requires more caution and attention from facilitators to take measures aimed at maintaining the confidentiality of group participants (2).

In the context of the COVID-19 pandemic, interpersonal relationships became predominantly remote, requiring adaptations at a time marked by uncertainties, these include: relationships mediated by screens can increase or decrease interpersonal connections; there may be a loss of control over the online space, as the facilitators no longer have control over the physical space occupied by the group participants, which may impact on confidentiality; and limited bodily interactions and difficulty in perceiving, understanding and responding to verbal and non-verbal signals presented by group members (30, 35).

The cohesion of virtual clinical listening groups was worked on from the alignment of the objectives of the experience with each participant determined from the contact prior to the groups, as well as through the investment in the emotional involvement between the students from the beginning and during the group meetings. Cohesion is one of the pillars of group work and is directly related to the empathy that its participants can develop in relation to the facilitators and other group members (30). Thus, in the virtual clinical listening groups, the bond between the participating university students was being woven and mixed with the feeling of empathy, keeping them united and helping them to deal with the challenges they needed to deal with, despite the absence of body-to-body interaction, considered one of the main obstacles in the realization of online groups (35).

Relationships with one's own body and with the bodies of other people have undergone transformations, faced especially with the new medias, which have mostly been generating the method of contact with the world at a time of social distancing, as has occurred during the pandemic of COVID-19 (7, 27). The bodies, in this scenario, do not appear in full, but in a partial or cropped way, at the same time facial expressions can be observed in an amplified way, through the approximation of the screen (32). In this context, facial expressions can be better observed online through the perception of changes, both in speech intonation and in subtle facial movements that reflect attitudes toward shared sensations and feelings (31).

In the virtual clinical listening groups, the facilitators faced the challenge of developing a comprehensive look at the variability of the expressions of the different participating students arranged in front of their computer screens or smartphones. It was essential for the facilitators to work in pairs in order to enhance their perceptions and, when students were available, to invite them to express how they felt physically and emotionally. Eventually, the need for better framing of the screens was also asked of the students, when they were shown to be too far away or too close to the camera to the point of appearing cropped. In these interventions, the facilitators were careful to highlight to the students both the importance of the quality of bodily presence and emotional availability for the encounter. When the student turned off the camera, got up frequently, turned off the audio to talk to someone who was physically close, or when their image appeared unclear or shaky, for example, the facilitators checked how they felt and if they would like to share with the group what had happened to them.

Due to the current need for increasingly frequent online interactions, training in future skills and abilities can be especially useful for group facilitators, with the aim of increasing their sensitivity and their ability to perceive changes in participants' facial expressions and being able to establish an alliance with them in order to increase group cohesion (31). Most psychologists are not usually skilled in the use of technology for virtual care and, like some of their patients, many are uncomfortable using it, often making the technology itself the most significant barrier in its use in the psychological clinic (32). However, Sansom-Daly and Bradford (31) believe that an entirely new intimacy can arise when connecting with another person through their smartphone or computer, even from their home space. Interpersonal contact, although with modifications, does not lose the possibility of forming a bond and being a source of help, which was highlighted by the students of the virtual clinical listening groups, stating that the online meetings were an important source of support, helping them to recognize what they felt and discover how they could deal with what was happening to them, using the technological and interpersonal resources available.

The importance of care in the shared online space was highlighted through the recommendation that students participating in virtual clinical listening groups need to find a physical environment in which they feel protected to share their experiences in safety. The care for the physical environment expresses the facilitators' concern with the participants' privacy, which also involves the management of group dynamics, because, as the control of online interaction is more difficult than in face-to-face contacts, it also becomes the responsibility of the other participants in the groups (31). In addition to promoting greater security and confidentiality, taking care of the physical space of the students' place of connection allows for the development of mutual trust and the opportunity to facilitate the expression of themes that are more difficult to be shared (32). In this sense, the group facilitators realized that the students welcomed their guidance, given that most were comfortable with the chosen environment to connect with the groups in which they participated.

In online psychological listening, it seems to be even more necessary to focus on the experience and emotions experienced during the realization of the virtual clinical listening groups rather than emphasizing thematic content, this is to enable more intimacy in the relationships between the various university students participating in the groups (33, 34, 36). In the virtual clinical listening groups, the facilitators helped to cultivate group cohesion by promoting interpersonal interaction among students, focusing on what was experienced at the present moment of the meeting, which was a driving force for the formation of bonds capable of favoring speaking and mutual listening between students.

### Stress as a Condition of Psychosocial Vulnerability

During the virtual clinical listening groups, the university students shared that they felt vulnerable and that they also were aware of people near them because of the fear of possible contamination and the need for social distancing, which generated intense emotional effects, with negative repercussions on their mental health. Some studies, such as those by Schmidt et al. (9) and Zhou et al. (39), indicate that symptoms of anxiety, depression, and stress have been the most mentioned in the experience of people during the COVID-19 pandemic, and are manifested as physical and mental exhaustion due to greater demands for productivity at work, interruption of dreams and projects, difficulty in adapting to the remote modality in their contact with others, crises in family relationships, unemployment, financial problems, and mourning the death of loved ones.

Among university students, the manifestations of psychological suffering have also increased significantly (5, 6). In the university context, the little attention paid to mental health by students can impact their academic activities, generating negative results in their professional construction and the development of psychopathological experiences (6, 39). Participants in the virtual clinical listening groups said they learned less in remote classes, found it difficult to pay attention to academic activities, and missed socializing on the University campus. The damages due to the physical distance from the University space were highlighted more frequently by newly enrolled students, graduates and those who were entering the curricular internship, which indicates that it is in moments of transition of the academic path that face-to-face interaction seems to gain greater relevance. The long-term impacts of periods of stress experienced due to prolonged physical distancing due to the pandemic are not yet clear, as the social support received is one of the protective factors against the development of psychopathological experiences (28).

As a way of coping with stressful situations, people tend to seek help from other people in order to obtain information that can reassure them as well as emotional support in face of the difficulties experienced as threatening (37). Under stress and alone, it may be more difficult to properly perceive which situations or actions are safe and to recognize what is necessary for a reduction in the sense of threat to occur, as is currently the case, with the risk of contamination by the CORONAVIRUS (36). In this sense, the offer of virtual clinical listening groups during the COVID-19 pandemic by the University was perceived by the students as an important care action for them.

Considering that the academic context can be normally stressful, constituting a space that is sensitive to psychosocial risks with regard to mental health (6), the virtual clinical listening groups promoted the development of a space for speaking and welcoming the demands of students, commonly associated with anxiety, stress and project uncertainty in view of the consequences of the pandemic. According to Zhang et al. (41), interventional actions in mental health care, even if remotely, are important because they address the stressors related to the pandemic and the adaptation difficulties imposed on people during this period, which, specifically in the educational experience, can impact academic performance, learning, and the continuation of students at the University (40).

The facilitators of the virtual clinical listening groups welcomed the most varied speaking demands, not restricting to academic complaints, as they understood that the life experience as a whole was potentially impacted by the COVID-19 pandemic crisis. The displacement of the educational environment into domestic spaces was felt by some students participating in the groups as a producer of instability in family relationships, as their homes were not previously prepared to function as places of study, work and leisure. Likewise, Schmidt et al. (9), highlight that social distancing and restriction, as well as changes in routines, brought harm to mental health and psychological wellbeing, being drivers of stressful experiences.

### Caring and Being Cared for in the Context of the COVID-19 Pandemic

The attention and care among the participants of the virtual clinical listening groups were pre-dominantly perceived in the movements built in the group dynamics. As they felt welcomed and cared for, the students began to offer a welcoming listening to their colleagues, which was also encouraged by the facilitators, seeking to ensure that interactions were not limited to a dual relationship within the groups and that the dialogue between the participating university students were a source of self-care and mutual care and that it was experienced as a fertile field for affective exchanges. Sansom-Daly and Bradford (32) emphasize that, in the online modality, psychologists need to be more attentive to the genuine presence, to the needs of people and to the distractions of contact with the experience of the moment than those generated by the stimuli of their computers. During the realization of the virtual clinical listening groups, the facilitators were attentive to the here and now of the participating students, inviting them to experience in a group in order to encourage them to get in touch with what they would like to share with others.

Virtual clinical listening groups, as a set of interventional actions aimed at university students, highlighted the need for attention and care to the psychological effects of the pandemic, given that such impacts can extend over time, reinforcing the importance of continuity of the existence of online psychological listening spaces in the context of Universities (11). Such social value was recorded in the statements of the group participants, highlighting the importance of discussion and mental health care at the University and suggesting that the groups of virtual clinical listening could continue in a post-pandemic future, positioning the promotion and prevention of mental health as a constant agenda in the practices and services developed for the students.

The limited offer of spaces aimed at prevention and online health treatment in Universities, at least before the COVID-19 pandemic, may have made more students less willing to seek virtual groups as spaces for psychological listening and care with mental health. On the other hand, because university students are usually familiar with technology in their academic routine, the offer of virtual clinical listening groups may have facilitated their participation in the groups. Marmarosh, Forsyth, Strauss and Burlingame (38) state that online groups, in the period of the COVID-19 pandemic, have been used with different audiences, such as health professionals and people experiencing grieving processes, in order to provide care for their physical and emotional health, as psychosocial interventions seem to have an impact on their immune system, thus increasing their importance as preventive actions in health care. Noteworthy among the disadvantages identified in online psychological care are: the limitation of the relationship in virtuality, as the psychotherapist does not see the virtual clinical listening group participants completely, but primarily their faces only; the difficulty in carrying out experiments, especially dynamic ones, and the inadaptability of other resources to the online context; the absence of bodily responsiveness; and difficulties in ensuring confidentiality and handling emergency situations. As such, online interventions can demand more care on the part of the facilitator, particularly when they involve group work (32). Such obstacles were seen in the virtual clinical listening group interventions and were minimized through prior organization and training of the research team, from their structuring to the interventions being conducted by more than one psychologist involved in the facilitation of each group.

The pandemic generated new challenges in health care, which could gradually change the scenario of those seeking these services. Virtual care seems to present itself as an increasingly frequent possibility in a future post-pandemic situation, as many started to have their first contact with health care during the period of social distancing. The technology has already been used both as a means of expanding the public to be cared for and as a resource for expanding the professional's presencial capacity in terms of support and safety for their patients, regardless of their location. However, with this new possibility, there may be more demands on the professional regarding the responsibility for managing crises, which requires further studies regarding the limits and conduct of interventions in the online care modality (36).

The use of online practices, such as telemedicine (34) and other forms of virtual care, such as clinical listening groups, can be hindered by concerns about privacy and technological barriers (32). Such practices will tend to remain and gain more space in a post-pandemic future, including in Universities, important institutions both in providing health services and in building knowledge about how group interventions can facilitate and promote health in times of crisis through the use of online technology. Fear and the feeling of helplessness, added to human and economic losses, will continue to demand attention to the harmful effects on health from professionals in the area (36). It is a process of building knowledge about how virtual clinical listening groups, in the long term and in times of crisis, can function as good practices aimed at providing psychological support to different audiences (39).

With increasingly available technologies, it is possible to reach larger populations, as well as to increase the amount of prevention and health treatment work in the form of virtual groups. The experience with virtual clinical listening groups points to a fertile field in healthcare in the context of universities. The groups carried out with university students from different courses and age groups, allowed for different interactions, reached a greater number of people than the individualized practices and promoted spaces in which each participating student could be cared for not only by the group facilitators, but also by the other students, strengthening a circularity that characterizes the therapeutic potency of this meeting format.

## Conclusion

The virtual clinical listening groups with university students were characterized as spaces for psychological intervention focused on the present experience, promoting a relationship of mutual care through speaking and listening to the participants' demands, which were mostly associated with anxiety, stress and uncertainty regarding to their life projects and expectations for the future, in view of, above all, the experiences of the consequences of the COVID-19 pandemic. They functioned as collective spaces for the growth of the participants, giving them personal and relational support, and expanding the expression of their feelings and their openness to their own experiences and to those of others while dealing with their common experiences, as well as their particular ways of coping with the pandemic. In the virtual clinical listening groups, there was a strengthening of the university students' self-Support as they realized that they were not alone and that other people also experienced similar difficulties.

The suffering experienced in the context of the current COVID-19 pandemic was the main motivation for university students to participate in virtual clinical listening groups, especially in the months of greater restriction to daily activities of study, leisure and work, with stricter social distancing in the period between September and November 2020, when the groups were held. These groups provided opportunities to share experiences and listen to how other university students experienced them, being able to exercise important social skills, devising new ways of coping to deal with the psychological impacts caused by the context of crisis and acting from a preventive and promoting perspective of mental health.

The group bond was built from the experience of the feeling of empathy and the feeling of security that the shared experiences would be kept confidential. The facilitators helped the students to cultivate group cohesion by promoting interpersonal interaction, focusing on what was experienced in the group's present moment, which favored cooperation between the participants.

For the facilitators of the virtual clinical listening groups, it was a great challenge to be limited but attentive to the participants' facial expressions as displayed on the screens of computers or smartphones. However, working in pairs enhanced perceptions, as well as attention to framing in the different screens. It was important that the facilitators were aware of the here-now of each participant, inviting them to experience the groups so that they could get in touch with the experiences they would like to share. The group facilitators sought to ensure that interactions were not only centered on dual relationships experienced within the groups, but also extending them to other participants so that the dialogue was experienced as a source of self-care and mutual care, constituting a fertile field for affective exchanges between the students.

Several university students shared that they learned less and that they found it difficult to pay attention in remote classes due to the lack of face-to-face interaction. Complaints about the physical distance from the University were cited more frequently by newcomers, graduates and those entering the curricular internship, which indicates that it is in moments of transition on the academic path that the lack of physical contact can be felt with the greatest impact. The change from the educational environment to the domestic space produced instability in family relationships as their homes were not prepared to be places of study, work and leisure all at the same time.

The difficulty of carrying out group meetings is highlighted in the face of some resistance from students. The lack of contact of students with their own mental health, as well as few opportunities for health care spaces carried out online prior to the COVID-19 pandemic, may have negatively influenced the willingness of more students to participate in the groups of virtual clinical listening. On the other hand, because university students are usually more familiar with technology, the virtual modality of groups may have been a facilitator for those students who actually participated in these groups. It is noteworthy that, among students, there was clearly positive feedback regarding the quality of the clinical listening experienced in these spaces and the stimulating effect on their mental health.

It is important to discuss and take care of mental health at the University. Mental health care through online groups has been presented as a possibility that is increasingly envisioned in the post-pandemic future.

Virtual clinical listening groups are a rich resource for the prevention of mental health problems and for the promotion of systematic care, developed and shared collectively for the benefit of university students. We realized that the virtual clinical listening group participants genuinely immersed themselves in the group experience, which can be seen identified in the fact that some returned for other groups offered at the university. Even with brief meetings and without continuity, that is a limit of the study, we were able to conclude that the group experience was able to promote care for mental health.

The group intervention revealed itself to be a more appropriate model in the pandemic situation for gathering more people who had lived a common pandemic experience. The experience with the virtual clinical listening groups provided an important mental healthcare resource in the context of the COVID-19 pandemic crisis.

We stress that the efficacy of this type of intervention was not directly investigated, as the methodological design of the study did not incorporate sufficient data to elucidate factors that might indicate significant changes over the course of the processes experienced by the virtual clinical listening group participants. As a consequence, the researchers were not able to gain broader insight into the possible long-term psychological impacts on the groups lead by them, what could be a subsidy for news interventions. We suggest undertaking new investigations with the aim of understanding more specifically which factors might be best worked, seeking to promote the mental health of the virtual clinical listening group participants.

## Data Availability Statement

The raw data supporting the conclusions of this article will be made available by the authors, without undue reservation.

## Ethics Statement

The studies involving human participants were reviewed and approved by University of Fortaleza Ethics Committee. The patients/participants provided their written informed consent to participate in this study.

## Author Contributions

LB, JA, JL, SB, and KC: conception, planning, and analysis or interpretation of data. JA and LB: writing of the article or its critical intellectual review. AM, GB, and VM: responsibility for final approval for publication. All authors contributed to the article and approved the submitted version.

## Funding

The COVID-19 public notice, launched by the Edson Queiroz Foundation through the Research, Development and Innovation Directorate (DPDI), is yet another action developed by the institution to fight the pandemic. In all, the institution has invested around R$400 thousand in 10 projects developed by its team of researchers in the areas of mental health, virological aspects, gender violence in social isolation, and preventative actions in public health, among others.

## Conflict of Interest

The authors declare that the research was conducted in the absence of any commercial or financial relationships that could be construed as a potential conflict of interest.

## Publisher's Note

All claims expressed in this article are solely those of the authors and do not necessarily represent those of their affiliated organizations, or those of the publisher, the editors and the reviewers. Any product that may be evaluated in this article, or claim that may be made by its manufacturer, is not guaranteed or endorsed by the publisher.
